# Awake versus asleep deep brain stimulation targeting the caudal zona incerta for essential tremor

**DOI:** 10.1038/s41531-024-00833-9

**Published:** 2024-11-22

**Authors:** Rasmus Stenmark Persson, Yulia Blomstedt, Anders Fytagoridis, Marwan Hariz, Patric Blomstedt

**Affiliations:** 1 Department of Clinical Science, Neurosciences, Umeå University, Umea, Sweden; 2 Department of Public Health and Clinical Medicine, Umeå University, Umea, Sweden; 3 Department of Clinical Neuroscience, Neurosurgery, Karolinska Institute, Stockholm, Sweden; 4 UCL Queen Square Institute of Neurology, London, UK

**Keywords:** Movement disorders, Neurological disorders, Parkinson's disease

## Abstract

To compare awake and asleep deep brain stimulation (DBS) surgery for Essential Tremor (ET), we conducted this retrospective cohort study of patients consecutively operated with DBS targeting the caudal Zona incerta (cZi). 37 underwent surgery awake and 55 asleep. Tremor before surgery and on/off stimulation one year after surgery were evaluated using the Essential Tremor Rating Scale (ETRS). Procedural time, electrode localization, stimulation parameters and adverse events were noted and compared. ETRS scores were similar at baseline between the groups except for contralateral arm tremor, which was slightly worse in the awake group. Total ETRS, contralateral arm tremor and activities of daily living scores showed no significant difference between the groups on-stimulation at one-year follow-up. Compared to the awake group, the asleep group had shorter procedural time and lower stimulation parameters. There were no intracranial haemorrhages nor surgery site-infections. Both groups showed a good improvement of tremor at one-year follow-up. Image-guided DBS surgery targeting the cZi enables safe and efficient asleep surgery for ET.

## Introduction

Medication-refractory essential tremor (ET) is a debilitating disorder, which can be treated with Deep brain stimulation (DBS). Traditionally, DBS surgery is performed awake to allow for intraoperative test stimulation, sometimes with concurrent microelectrode recording, to identify the physiological target and evaluate the effects and side effects of stimulation. However, with the advances in MRI-based visual anatomical targeting and image-verified surgery, there has been a clear trend towards DBS surgery under general anaesthesia, so called “*asleep* DBS”.

Most publications reporting on asleep DBS have concerned surgery targeting the subthalamic nucleus (STN) and the globus pallidus internus (GPi) in patients with Parkinson´s disease or dystonia^1–6^. Here, a number of non-randomized studies have found the effects of awake and asleep surgery to be comparable, at least in terms of motor outcome^7–12^. Recently two randomized trials of awake versus asleep surgery have demonstrated non-inferior motor outcomes after asleep STN-DBS for PD^13,14^.

Both the STN and the GPi are readily and easily visualized on conventional MRI, which is not the case regarding the third commonly used target for DBS, the ventral intermediate nucleus of the thalamus (Vim). This is probably one of the most important reasons as to why there has been more reluctance to perform asleep DBS for ET. Only recently have a couple of studies been published on asleep DBS for patients with ET^15–17^.

We have previously reported that by targeting the caudal Zona incerta (cZi) within the posterior subthalamic area (PSA), it was possible to alleviate the tremor up to at least 10 years after surgery^18,19^. The cZi and PSA are located between two structures readily visible on MRI, i.e., the red nucleus and the STN. This allows for a more direct targeting than the Vim, and might hence be a more suitable target for asleep DBS. In 2004 as part of a prospective open-label study, we started to target the cZi instead of the Vim for DBS in patients with ET^19^. Here, we present our experience of awake and asleep cZi-DBS for ET.

## Results

A total of 96 consecutive patients were implanted with 113 electrodes for ET. Ten patients were excluded from the present analysis: three patients had mislabelled or re-evaluated diagnosis; in one patient, the electrode was implanted in using Vim as a surrogate target and electrode location in the PSA could not be verified due to missing MR-images; six patients had missing one-year data, one due to ipsilateral stroke and one due to DBS extirpation because of an inflammatory reaction to the electrode. One patient with hereditary, alcohol-responsive bilateral action tremor for four decades was implanted on two separate occasions (asleep) and had developed parkinsonian symptoms by the time of the second implantation. The outcome of the second electrode was excluded from analysis, resulting in 102 electrodes in 86 patients with available one-year results.

Patients without one-year data were still included in the adverse event analysis. Of the 86 patients, sixteen (5 awake/11 asleep) had bilateral procedures, resulting in 40 electrodes in the awake group and 62 electrodes in the asleep group.

The primary outcome was changes in the Essential Tremor Rating Scale (ETRS) between baseline and the on-stimulation condition at one-year follow-up. Patients with bilateral DBS were evaluated concerning one body side at a time with the stimulation on the other side turned off. We performed a comparison between awake and asleep surgery. The secondary outcomes were differences between the groups regarding procedure time (from the first incision until the closure of the last incision after implantation of electrodes, connection cables and implantable pulse generator), active contact location, stimulation parameters, and adverse events. Adverse events included all 92 patients with ET having been implanted with cZi as a target.

Demographics and patient characteristics are shown in Table 1. There was a tendency towards older patients with longer disease duration in the asleep group, but no variable was significantly different between the groups (*p* > 0.05).

**Table 1:** Patient characteristics of the whole cohort and subgroups

	All	Awake	Asleep	vs *p*
Patients	86	35	51	
Electrodes	102	40	62	
Unilateral/Bilateral	70/16	30/5	40/11	
Female/Male	34/52	14/21	20/31	
Age at onset	36.2 ± 19.2 (7–69)	38.2 ± 21.4 (7–69)	34.9 ± 17.7 (7–63)	0.233
Age at surgery	65.7 ± 1.6 (25–80)	63.8 ± 14 (25–80)	66.9 ± 7.5 (45–79)	0.452
Disease duration	29.2 ± 18.2 (4–69)	25.4 ± 17.3 (4–69)	31.8 ± 18.5 (4–68)	0.104
Heredity Yes/No/Unknown	60/22/4	24/9/2	36/13/2	
Alcohol response Yes/No/Unknown	55/6/25	26/2/7	29/4/18	
Handedness right/left	81/5	32/3	49/2	
Side of electrode R/L/Bilateral	7/63/16	5/25/5	2/38/11	
Electrode model 3387/3389	25/77	25/15	0/62	
Follow-up (months)	12.3 ± 1.8 (11–24)	12.7 ± 2.8 (12–24)	12 ± 0.3 (11–13)	

Expressed in numbers or mean ± SD (min-max). Welch’s T-test. R = right side. L = left side.

Comparing ETRS-scores at baseline revealed a slightly worse contralateral arm tremor scores in the awake group than in the asleep group (*p* = 0.01). Total ETRS, Part A (items 1–9), sub-items postural/action-tremor, hand function, and activities of daily living (ADL) scores showed no difference between the groups at baseline.

### Whole cohort

All values are expressed as mean ± standard deviation (SD) and concern the effects of unilateral stimulation unless stated otherwise.

**Fig. 1:**
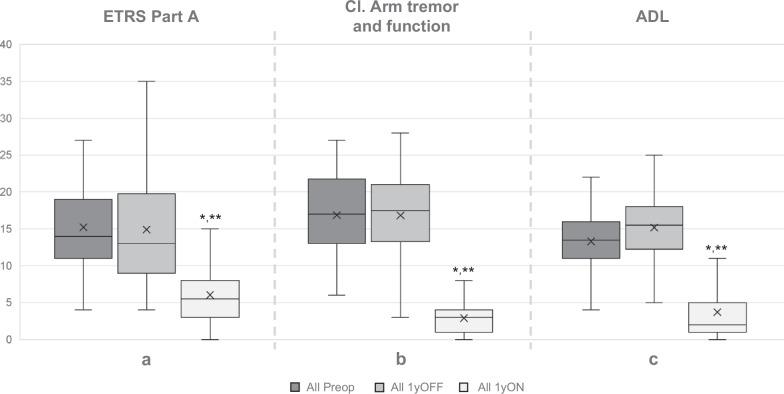
Boxplots of ETRS subitems for the whole cohort. **a** Part A (tremor items 1-9). **b** Compound score of contralateral (Cl.) arm tremor and function (Cl. Items 5/6 + 11-14). **c** Activities of Daily Living (ADL, items 15-21) score. Y-axis = ETRS score. x = mean. 1yOFF = off stimulation at one-year. 1yON = on stimulation at one-year. *p < 0.001 vs baseline, Wilcoxon signed rank. **p < 0.001 vs off stimulation at one-year follow-up, Wilcoxon signed rank.

Total ETRS improved from 51 ± 1 points at baseline to 21.1 ± 12.0 on stimulation at one-year follow-up with a mean individual percentual improvement of 58.9% (*p* < 0.001, Wilcoxon signed rank). At follow-up, total ETRS improved by 60% with stimulation from a score of 53.2 ± 17.4 off-stimulation. Selected subitems are presented in Fig. 1a–c.

Contralateral arm tremor was improved from 5.9 ± 2.2 at baseline to 0.3 ± 0.7 on stimulation at follow-up (*p* < 0.001). Specifically, 96 evaluated arms had a score of 0–1 (78 arms with 0 points and 18 with 1 point), 4 arms had 2 points and 2 arms had 3 points. Regarding action tremor, only one patient had a score >1 (2 points, awake group).

A compound score of contralateral arm tremor and function (items 5/6 and 11–14) was significantly improved from 18.0 ± 5.5 at baseline to 3.2 ± 3.5 on stimulation at follow-up. ADL improved from 13.3 ± 4.1 to 3.7 ± 4.5 (*p* < 0.001). At follow-up, the compound score was improved from 18.0 ± 6.7 off stimulation while ADL was improved from 15.2 ± 4.9 off stimulation compared to on stimulation (*p* < 0.001).

### Awake versus Asleep

There was a significant difference in procedure time (skin-to-skin, implantation of electrode, connection cables and implantable pulse generator) between groups, with unilateral asleep surgery being 1 hour and 39 minutes shorter than awake surgery on average ( ± 15 min Standard Error difference, *p* < 0.001, Welch’s t-test). Bilateral implantations showed a mean of 5.6 ± 0.6 hours for awake surgery and 2.9 ± 1.0 hours for asleep surgery (*p* = 0.002, Mann-Whitney U test was used due to small sample size failing assumption of normality).

Details of ETRS scores are available in Table 2. Total ETRS off-stimulation scores deteriorated in the asleep group from 50.8 ± 14.8 to 56.3 ± 15.4 points between baseline and one-year follow-up (*p* = 0.001, Wilcoxon signed rank). This was mainly due to worsening of contralateral hand function and ADL scores (*p* = 0.029 and *p* < 0.001, respectively). In contrast, the awake group tended to have better ETRS-scores at one-year follow-up in comparison to baseline, but the difference was not statistically significant except regarding contralateral arm tremor (*p* = 0.019).

**Table 2:** ETRS-scores at baseline and at one-year follow-up

	Max	Preop			1 year OFF stimulation			1 year ON stimulation		
		**Awake**	**Asleep**	***p***	**Awake**	**Asleep**	***p***	**Awake**	**Asleep**	***p***
**Total ETRS** It. 1-21	**144**	51.0 ±15.2	50.8 ±14.8	0.850	48.4 ±19.4	56.3 ±15.4	0.029	21.5 ±12.9	20.9 ±11.4	0.921
Min-max		27–93	25–101		9–89	29–103		0–70	1–73	
**Part A** It. 1-9	**80**	15.7 ±7.1	15 ±7.0	0.638	14.6 ±8.6	15.1 ±7.1	0.455	6.5 ±4.5	5.8 ±3.6	0.690
Min-max		5–37	4-47		4–38	4–42		0–18	0–16	
**Voice tremor** It. 3	**4**	0.6 ±0.9	0.9 ±1.1	0.101	0.4 ±0.8	0.7 ±1.1	0.169	0.2 ±0.4	0.3 ±0.6	0.174
Min-max		0–3	0–4		0–3	0–4		0-2	0-3	
**Head tremor** It. 4	**8**	0.6 ±0.9	1.3 ±1.7	0.015	0.7 ±1.2	1 ±1.2	0.040	0.2 ±0.4	0.3 ±0.6	0.412
Min-max		0-3	0-7		0-6	0-5		0-1	0-3	
**IL. Arm tremor** It. 5/6	**12**	5.0 ±2.3	4.3 ±2.0	0.257	5.4 ±2.7	5 ±2.3	0.380	4.4 ±2.5	3.7 ±2.5	0.175
Min-max		1-9	0-9		0-11	0-10		0-10	0-10	
**CL. Arm tremor** It. 5/6	**12**	6.6 ±2.2	5.5 ±2.2	0.013	5.8 ±2.7	5.6 ±2.3	0.800	0.3 ±0.6	0.3 ±0.7	0.798
Min-max		2-12	2-12		1-12	0-12		0-2	0-3	
**CL. Rest tremor**	**4**	0.9 ±1.1	0.4 ±1	0.008	0.8 ±1.1	0.4 ±0.8	0.048	0 ±0	0 ±0.1	1.0
Min-max		0-4	0-5		0-4	0-4		0-0	0-1	
**CL. Postural tremor**	**4**	2.5 ±1.0	2.1 ±1.1	0.114	1.8 ±1.2	1.8 ±1.2	0.919	0 ±0.2	0.1 ±0.4	0.133
Min-max		1-4	0-4		0-4	0-4		0-1	0-2	
**CL. Action tremor**	**4**	3.3 ±1.1	3 ±1.1	0.164	3.3 ±0.9	3.4 ±1.1	0.380	0.2 ±0.5	0.2 ±0.4	0.88
Min-max		1-4	1-4		1-4	0-4		0-2	0-1	
**Handwriting** It. 10	**4**	1.7 ±1.2	2 ±1.1	0.063	1.7 ±1.2	2.2 ±1.4	0.059	0.5 ±0.7	0.7 ±1.0	0.715
Min-max		0-4	0-4		0-4	0-4		0-3	0-4	
**Hand function** It. 11-14	**32**	19.7 ±6.4	20.9 ±6.0	0.439	18.8 ±7	22.3 ±6.6	0.017	10.3 ±4.9	11.2 ±5.9	0.619
Min-max		9-32	11-32		2-32	2-33		0-22	0-32	
**CL. Hand function**	**16**	10.5 ±3.9	11.3 ±3.4	0.327	9.5 ±4.2	12.3 ±3.4	0.001	2.2 ±3.1	2.9 ±2.5	0.021
Min-max		4-16	4-16		0-16	3-16		0-15	0-16	
**IL. Hand function**	**16**	9.1 ±3.8	9.6 ±3.6	0.514	9.4 ±3.8	10.3 ±4.0	0.205	8.5 ±3.8	8.4 ±4.3	0.733
Min-max		0-16	2-16		0-16	3-16		0-16	0-16	
**ADL** It. 15-21	**28**	13.8 ±4.1	13 ±4	0.815	13.3 ±5.7	16.4 ±4.0	0.004	4.3 ±5.2	3.3 ±4.0	0.710
Min-max		4-25	3-19		0-25	8-28		0-24	0-20	

Expressed as mean ± SD, min-max. Mann-Whitney U test between groups

Wilcoxon signed-rank test within-group. *IL* Ipsilateral to DBS, *CL* Contralateral to DBS, *It* items.

Comparing scores off-stimulation between asleep and awake groups at one-year follow-up, total ETRS (*p* = 0.029, Mann-Whitney U), contralateral hand function (*p* < 0.001) and ADL (*p* = 0.004) were worse in the asleep group.

### Results of stimulation

**Fig. 2:**
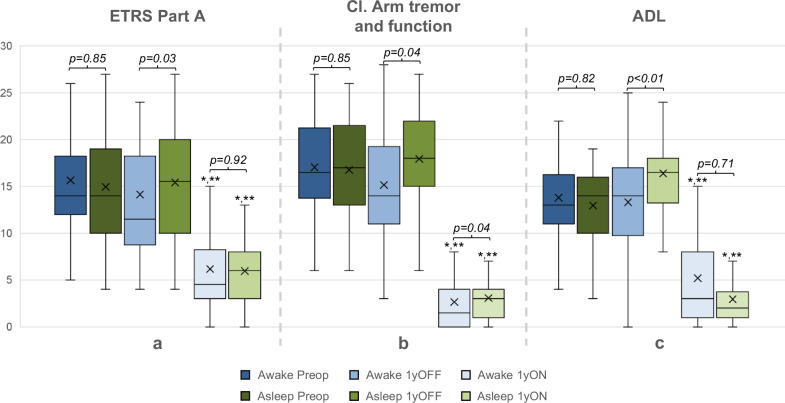
Boxplots of ETRS subitems in both groups over time. **a** Part A (items 1–9). **b** Compound score of contralateral (Cl.) arm tremor and function (Cl. Items 5/6 + 11–14). **c** Activities of Daily Living (ADL, items 15–21). Y-axis = ETRS score. *p* values above brackets = between groups, Mann-Whitney U. x = mean. *p < 0.001 vs baseline, Wilcoxon signed rank. **p < 0.001 vs off-stimulation at one-year follow-up, Wilcoxon signed rank.

Unilateral stimulation significantly improved mean total ETRS in comparison to baseline by 58% in the awake and 59% in the asleep group (*p* < 0.001, Wilcoxon). There was no significant difference in total ETRS between the groups on stimulation (*p* = 0.92). The compound score of contralateral arm tremor and function was significantly improved by stimulation in both groups, both in comparison to baseline and compared to off stimulation (*p* < 0.01, Fig. 2a). The scores on stimulation were similar between the groups regarding Part A and ADL scores (see Fig. 2b, c). The awake group had lower contralateral arm tremor and function scores on stimulation than the asleep group (*p* = 0.04). Comparing the separate subscores revealed similar contralateral arm tremor scores on stimulation (*p* = 0.79*)* between the groups but lower contralateral hand function scores in the awake group at follow-up (*p* = 0.021).

We then defined a poor response as <40% improvement on total ETRS (-1 SD) between baseline and on-stimulation at follow-up. There were 3 evaluated hemi bodies in the awake group with a poor response and 8 in the asleep group (non-significant, *p* = 0.521, Pearson Chi-square).

### Electrode location and stimulation parameters

In the awake group, intraoperative stimulation prompted 15 electrodes to be advanced 1–2 mm deeper during surgery. In the same group, one electrode was relocated during surgery using a second electrode pass. Further, in three patients a second electrode was implanted in the same session at a new location (within the PSA), while retaining the first electrode, due to an ambiguous stimulation response. This occurred in our early experience and in the end, the initial electrode was in all patients found to be more optimally placed and to provide a better effect and used for chronic stimulation. In the asleep group none of the electrodes had to be relocated following postoperative imaging verification.

**Fig. 3:**
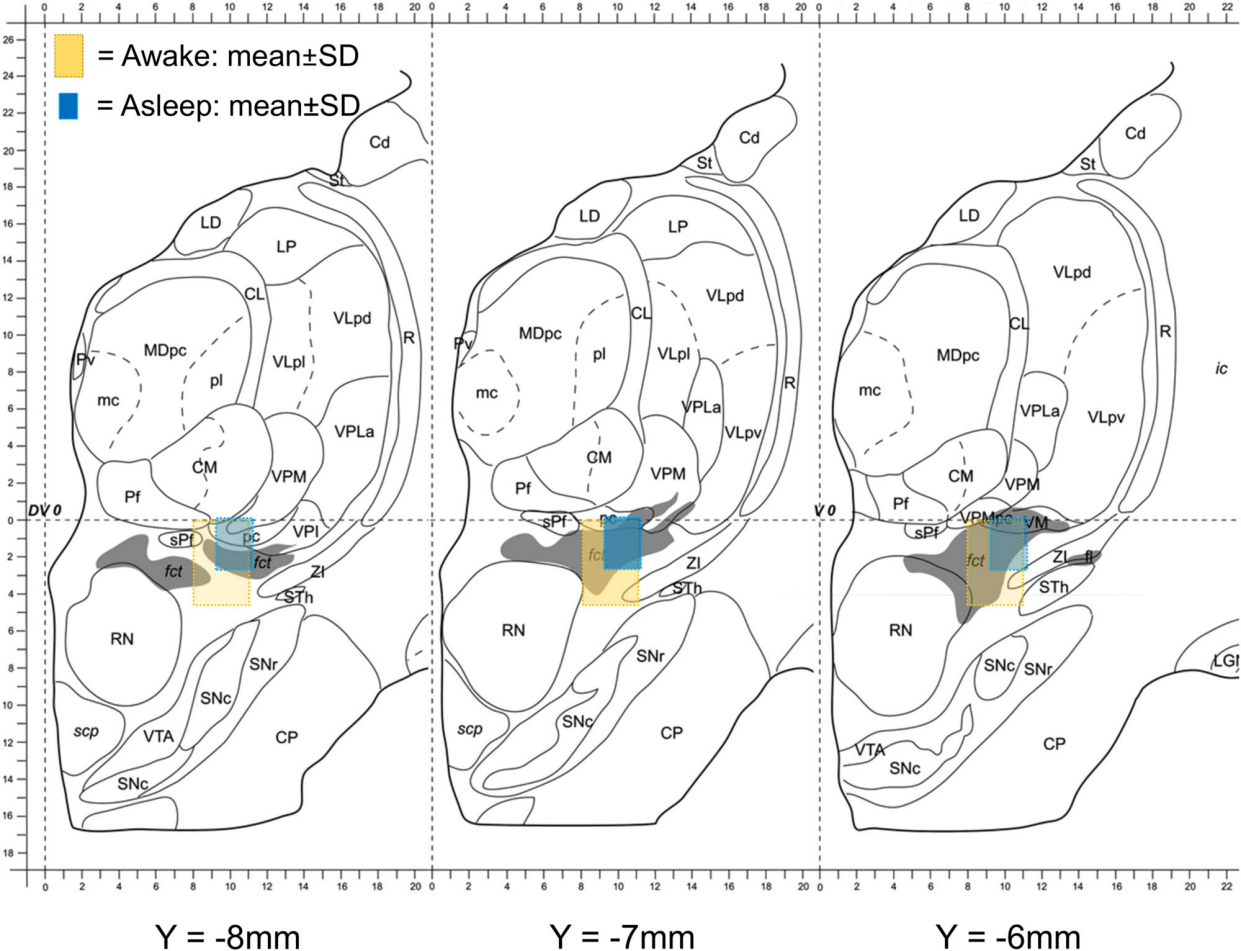
Mean location of the active cathodic contact in relation to the posterior tail of the STN (pSTN). Borders of the boxes represent ± SD of mean contact location in the medio-lateral and superior-inferior axis. Plotted on serial coronal slices of the Stereotactic Atlas of the Human Thalamus and Basal Ganglia by Anne Morel, Copyright © 2007. Reproduced by permission of Taylor and Francis Group, LLC, a division of Informa plc.

The mean location of the active cathodic contact in relation to the midcommissural point (MCP) and the posterior tail of the STN (pSTN) are presented plotted on coronal slices of Morel’s atlas in Fig. 3 and presented together with the most distal contact in Table 3. The most distal contacts, as well as the active cathodic contacts were located slightly more superior and more lateral in the asleep group than the awake group in relation to the pSTN. The awake group had a larger variance of the electrode location, as measured by the coordinates of the deepest contact, in laterality and in the anterior-posterior direction than the asleep group in relation to the pSTN but not in relation to the MCP (see Table 3).

**Table 3:** Location of the active cathodic contact and the deepest contact (“0”) in relation to the midcommissural point (MCP) and in relation to the posterior tail of STN (pSTN)

	MCP			pSTN		
	Active X	Active Y	Active Z	Active X	Active Y	Active Z
**Awake**	11.7 ± 1.6	−6.4 ± 1.6	−2.4 ± 2.2	−2.5 ± 1.5	−0.1 ± 1.3	1.8 ± 2.3
**Asleep**	12.3 ± 1.5	−6.3 ± 1.2	−1.0 ± 1.6	−1.8 ± 1.0	0.3 ± 1.0	3.0 ± 1.4
***p, Welch’s***	0.77	0.66	<0.001	0.009	0.111	0.005
	**X0**	**Y0**	**Z0**	**X0**	**Y0**	**Z0**
**Awake**	10.8 ± 1.3	7.8 ± 1.4	−5.3 ± 1.1	−3.4 ± 1.3	−1.4 ± 1.3	−1.1 ± 1.2
**Asleep**	11.3 ± 1.3	7.8 ± 1.1	−3.7 ± 1.1	−2.8 ± 0.9	−1.3 ± 0.7	0.3 ± 1.0
***p, Welch’s***	0.06	0.99	<0.001	0.005	0.50	<0.001
***p, Levene’s test***	0.12	0.63	0.60	0.03	0.01	0.06

Expressed as mean mm ± SD. Welch’s T-test. Negative values are medial to, posterior to, or below the reference point.

The mean difference was 1.2 ± 0.4 mm (± SE difference *p* = 0.005, Welch t-test) and 0.7 ± 0.3 mm (*p* = 0.009) regarding depth and laterality of active contacts, respectively. The only statistically significant difference regarding contact coordinates in relation to MCP concerned the depth (1.4 ± 0.4 mm deeper in the awake group, *p* < 0.001).

Mean stimulation parameters are available in Table 4. The awake group had higher amplitude settings (mean difference 0.37 V ± 0.14 SE difference, *p* = 0.008, Welch T-test*)* as well higher frequency (mean difference 15.1 Hz±3.6 SE, *p* < 0.001) and higher Pulse Effective Voltage (PEV, 0.05 V ± 0.01 SE, *p* < 0.001) than the asleep group.

**Table 4:** Stimulation parameters at one-year follow-up

	All	Awake	Asleep	*p Awake vs Asleep*
**Monopolar/bipolar**	90/12	30/10	60/2	
**Amplitude (V)**	1.99 ± 0.65	2.2 ± 0.7	1.85 ± 0.5	0.008
**Pulse width (µs)**	62.6 ± 10.4	64.5 ± 12.8	61.5 ± 8.5	0.188
**Frequency (Hz)**	155.2 ± 18.2	164.4 ± 19.6	149.3 ± 14.6	<0.001
**Pulse effective voltage (PEV)**	0.19 ± 0.07	0.23 ± 0.08	0.18 ± 0.05	<0.001

Expressed as mean ± SD. Welch’s T-test

### Adverse events and side effects

The adverse events are compiled in Table 5. There were no intracranial haemorrhages or infection necessitating removal of the hardware. One patient in the asleep group experienced gait disturbance, dizziness, and headache during the first 2 weeks after surgery. Imaging revealed significant peri-electrode oedema without contrast-enhancement and the patient had neither fever nor infection parameters. Extirpation of the electrode led to full recovery and the reaction was considered to be due to the inflammatory reaction around the DBS electrode. Another patient in the asleep group developed minor pulmonary embolisms three days after surgery. In the awake group one patient experienced sudden mild hemiparesis three days after surgery and concurrent pneumonia. The weakness improved but persisted to some degree five months later and was deemed be due to a lacunar ischemic stroke.

**Table 5:** Adverse events and side effects

**Surgical events**	
**Awake**	**Asleep**
4 intraoperative electrode repositioning/extra electrode	1 electrode extirpation due to inflammation
2 vasovagal responses	
2 severe fatigue	
1 unable to perform awake	
**Other perioperative events**	
1 lacunar infarction	1 pulmonary embolism
2 infections (pneumonia and urinary tract infection)	2 transient severe confusion
	1 drug reaction
	1 postop. fever without focal symptoms
	1 suprapubic catheter
**Total: 12 in 37 patients**	**Total: 7 in 55 patients**
**Adverse events within the first year**	
**Awake**	**Asleep**
4 minor microlesional side effects	4 minor microlesional side effects
3 straining cable/migrating IPG needing surgical adjustment	1 decreased hearing on one side
1 postoperative transient depression	1 increased seizure frequency
1 myocardial infarction 1 mo. postop.	1 transient global amnesia
	2 with increased fatigue/decreased condition
**Total: 9 in 37 patients**	**Total: 9 in 55 patients**
**Stimulation-induced side effects at 1 year**	
3 upper limb ataxia	6 upper limb ataxia
3 habituation	4 habituation
2 dysarthria	3 dysarthria
1 gait disturbance	1 gait disturbance
1 slight incoordination	1 paraesthesia
**Total: 10 in 40 electrodes**	**Total: 15 in 62 electrodes**

Regarding surgical events in the awake group, there were two patients who experienced a vasovagal reaction and two patients undergoing bilateral surgery who showed severe fatigue after first lead implantation which resulted in no intraoperative test-stimulation performed upon implantation of the second electrode. In one awake patient, mounting the stereotactic frame was impossible due to severe head tremor in combination with kyphosis. This patient underwent later asleep surgery.

Some patients experienced transient symptoms after surgery, such as headache, gait or speech disturbances that resolved within one month after surgery. Persistent microlesional side effects in the form of slight gait or speech disturbances at one-year follow-up off-stimulation were similar between the groups.

Clinically relevant habituation or upper limb ataxia necessitating multiple changes of the stimulation parameters were noted in 16 treated hemi bodies during the first year (6 awake and 10 asleep), with some experiencing both problems. One patient in the asleep group with upper limb ataxia did not want to decrease the parameters due to the otherwise beneficial effect on tremor.

## Discussion

This retrospective study analysed and compared the results of awake and asleep DBS targeting the cZi for ET. The symptom improvement was in line with previous publications on cZi-DBS for ET and showed that both awake and asleep surgery to be safe and efficacious in treating tremor^20–22^. Unilateral stimulation improved the mean total ETRS scores by 58% and 59% in the awake and asleep group respectively, while mean contralateral arm tremor was improved by 95% in both groups. No significant differences were seen between groups regarding Part A, contralateral arm tremor or ADL scores on stimulation at one-year follow-up. However, the subscore for contralateral hand function was higher in the asleep group on-stimulation. This could be due to the difference in hand function between the groups without stimulation, which was statistically significant at follow-up but not at baseline; or it might reflect the significantly lower stimulation strength in the asleep group.

Regarding the eleven ‘poor’ responders, one of three in the awake and four of eight in the asleep group had a clear response on contralateral scores ( > 70% on Cl. Arm tremor and function) but not the other ETRS items. In the awake group, all three “poor” responders had a good response on macrostimulation, and the electrode was not repositioned during surgery. In the asleep group, 6 of the 8 hemi bodies with a poor response belonged to bilaterally implanted patients. Among these, only one patient had a poor response on both sides, leaving 5 patients with one “good” side and one “poor” side. We found no difference in visualised lead localisation or response on monopolar review between the “good” and the “poor” side in these patients. This tendency of a larger deterioration of effect in bilaterally implanted patients is not unknown^18,23^ and has been suggested to be due to plasticity and lesser compensatory potential from an untreated side^24^.

To our knowledge, only one other group has specifically compared asleep and awake DBS for ET. Two separate studies by Chen et al.^15,25^ reported the results of Vim-DBS surgery using a coordinate-based targeting method. Of note, the patients were implanted awake or asleep based on the approval of the referring neurologist and the patient’s personal preferences. In the first retrospective study, Chen et al. sent out self-rated questionnaires at a mean of 17.7 months after surgery asking the patients how they would assess both their pre- and postoperative tremor, which may introduce a possible recollection bias. In addition, only 19 patients in the awake group and 11 patients in the asleep group responded to the questionnaire (48% and 65% responder rate respectively). In the second study, these authors measured quality of life and tremor using a smartphone accelerometer in addition to the self-assessment. In the awake group, questionnaire data from 11 patients and accelerometer data from 17 sides were available at 3-month follow-up. Correspondingly, data was available from 33 patients and from 48 sides in the asleep group. Notwithstanding some missing data, the authors found no difference in outcome between the awake and the asleep group in either study. As others have argued, a three-month follow-up period could obscure a difference in efficacy between the groups due to the sometimes long-lasting tremor-reducing effect of peri-electrode oedema, which is partly why we chose a longer follow-up in the current study^26,27^.

Regarding accuracy, the coordinates of the planned target were not available in this study, restricting the comparison of implantation accuracy to an interpretation of other parameters.

The active contacts were located more superior, and more lateral, to the pSTN in the asleep group than in the awake group. This difference can partly be explained by evolution of the surgical technique. During awake surgery, after macrostimulation, the electrode was implanted at a depth to ensure at least one contact being located below the intraoperatively established target level, as opposed to having the most distal contact centred at the target. In-house analyses showed that the most distal contacts were seldom the most effective. When transitioning to asleep surgery, the most distal contact was instead centred at the preplanned target. Due to the mediolateral angle of the trajectory, implanting the electrode less deep would also result in a more lateral location.

In addition, we found that there was a larger variance in X and Y-coordinates in relation to the pSTN in the awake group than the asleep group. This could be due to the relocation of some electrodes after macrostimulation. However, only four of the 35 patients had a repositioning or additional electrode after testing. In the end, a randomised trial, including deviation from planned target is needed to elucidate differences in accuracy.

Concerning stimulation parameters, the lower stimulation strength in the asleep group could be due to a more efficient localisation of the electrodes in the asleep group (under the assumption of equal efficacy). As the electric field strength is in relation to the distance from the active contact, having the contacts closer to the physiological target would limit the stimulation strength necessary to disrupt the pathological activity associated with tremor^28,29^.

On the other hand, lower stimulation strength could also be due to more stimulation-induced side effects in the asleep group during adjustments. However, bipolar stimulation, usually used to avoid side effects, was more common in the awake group which would indicate the opposite.

Finally, the difference in stimulation strength and mode might be explained by a change in the programming regime. During the first years of using cZi as a target for ET, we tried to maximise the efficacy of the stimulation on tremor. With experience came the notion of a higher current driving some side effects such as ataxia and/or rebound^30^. In addition, some side effects, such as gait disturbance in difficult terrain, were not apparent during monopolar screening and was only revealed over time. This resulted in a more holistic programming approach, trying to achieve a good tremor relief but keeping the stimulation strength as low as possible.

In summary, it is difficult to determine the definite reason behind these differences and as this was a retrospective study, it is wise to interpret these results with caution. In general, the energy consumption in cZi-DBS in this study seemed to be lower than what has been published for Vim-DBS^31–35^.

As the results from the GALAXY randomized trial of awake versus asleep STN-DBS for PD suggested^14^, asleep surgery is probably more convenient and less stressful for both patient and surgeon; A better quality of MRI images can be achieved since movement artifacts are abolished, possibly resulting in better targeting.

A completely horizontal position is uncomfortable for the patient in awake surgery but is not an issue in asleep surgery. Asleep surgery and a horizontal position might reduce the risk of venous air embolism^36,37^ and may also increase accuracy^38^; Shifting from a semi-sitting position used in patients undergoing awake DBS, to a horizontal position has in our experience greatly reduced leakage of cerebrospinal fluid (CSF), probably since the brain will press against the burr hole, thus sealing it.

An important aspect regarding CSF leakage is time, and several parts of the procedure are in our experience, faster with the patient asleep. Most importantly, asleep surgery allows the implantation of the extension cable and IPG to be done directly (without removing the frame). Shorter surgery may also decrease the risk of surgical site infection^39,40^. There is further an economical aspect with awake surgery being more expensive than asleep, especially when microelectrode recording is used^41,42^.

There are of course also drawbacks to asleep surgery: It does not allow for intraoperative evaluation of intraoperative stimulation effects and side effects. This lack of immediate feedback both regarding tremor reduction and side effects can lead to an electrode being implanted suboptimal in relation to the physiological target.

In addition, awake surgery with either test stimulation or microelectrode recording can significantly contribute to an increased understanding of the physiology of different anatomical structures and diseases^43^.

In the current study, there was often a pronounced microlesional effect with disappearance of tremor at the introduction of the electrode into the PSA. Sometimes the depth of the electrode, and rarely other coordinates, were adjusted following macrostimulation. The interpretation of macrostimulation response was sometimes difficult due to the disappearance of tremor due to this ‘stun effect’. However, stimulation was carried out to elicit side effects such as dysarthria, ataxia, and paraesthesia. The interpretation of these side effects can sometimes be bewildering, as we have previously described^44,45^, and most of them showed adaptation after seconds or minutes of intraoperative stimulation.

Still, it is possible to perform asleep DBS successfully, as has been done with GPi-DBS for over two decades, and more recently also with STN-DBS^7,10,46,47^. Common for these two targets is that they are easily identified with visual anatomical targeting on conventional MRI. This is also one of the main reasons why asleep atlas-based Vim-DBS surgery for ET has been met with more apprehension. Attempts have been made to visualize the cerebellothalamic fibres passing through the PSA into the Vim^48,49^, which is probably the pathway through which both “Vim”-DBS and “cZi/PSA”-DBS exerts its effects^50–52^. However, this technique is still in its infancy^53^.

Lately, the PSA/cZi has become increasingly common as an alternative target^54^and was in two randomized blinded trials demonstrated to be more efficient and/or more effective than Vim-DBS for tremor^27,55^.

One of the advantages of the cZi, especially with regard to asleep surgery, is that the target can be visualized on MRI^54^. Even though one cannot visualise the cZi as such, this target area is identifiable on an axial MRI at the level of the largest diameter of the Red Nucleus (RN), in between the RN and the posterior tail of the STN.

Certain limitations should be taken into account when considering the results of the current study. The major limitations are the lack of randomisation between asleep and awake DBS and the retrospective, unblinded nature of the study. Another possible limitation is that asleep DBS in our clinic was implemented after the surgeon had accumulated more than a decade-long experience in awake DBS, allowing for a documented in-depth knowledge of the anatomy and macrostimulation physiology of the target area and its surroundings. This may well have facilitated the confidence in implementing asleep DBS, without compromising the good results. In addition, the follow-up period was relatively short, and several studies have shown that the efficacy of both Vim-DBS and cZi-DBS can decline over time^18,24,32,33,56^. As such, differences in the development of loss of benefit after one year, so called *late escapers*^30^, or late-onset side effects cannot be deduced from this study. Finally, it must be acknowledged that the groups differed in the number of patients and a higher contralateral arm tremor score in the awake group at baseline.

In summary, asleep and awake DBS targeting the cZi were both found to be safe and effective treatments for patients with ET. Asleep DBS was associated with a shorter procedure time and lower stimulation parameters. Asleep DBS has some advantages worth striving for but should only be performed if the efficacy and the safety of the treatment can be maintained. The outcome of this study indicates that it is possible to perform asleep DBS for ET with good results using a proper targeting technique. However, as these results concern only one-year data, longer-term follow-up studies are needed to discern any differences over time. Ultimately, prospective randomized rater-blinded trials are needed to elucidate any differences in efficacy or adverse events between awake and asleep surgery.

## Methods

Since 2004, cZi has been used as the primary DBS-target for tremor at our unit. Initially, the surgery was done with the patient awake, but since 2011 all procedures have been performed under general anaesthesia. In the present study, we perform an analysis of the one-year outcome of patients implanted with constant voltage DBS-systems using awake or asleep surgery between 2004-2017. Some of these patients have been published previously in other contexts^18,19^. All procedures were performed by the same surgeon (PB).

The diagnosis of ET was set by a senior movement disorder neurologist prior to the updated tremor classification of 2018^57^ and the patients were referred to surgery after pharmacological treatment had been proved insufficient, intolerable, or unsuitable. Informed consent was obtained according to the Declaration of Helsinki and the study was approved by the Ethical Committee at the University of Umeå.

### Surgery

**Fig. 4:**
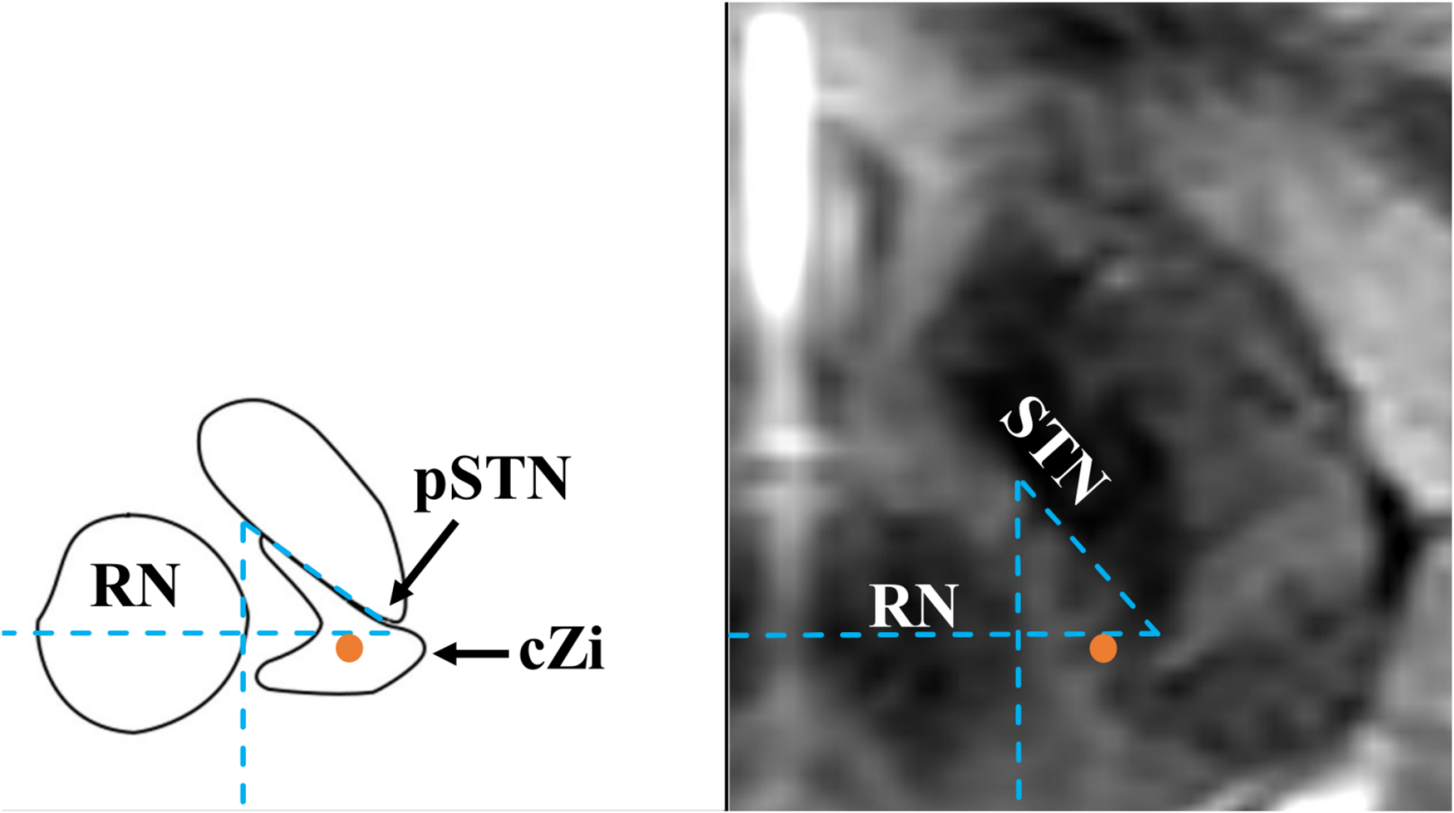
The target (orange marker) with mental helplines. As the relation between the red nucleus (RN) and the posterior tail of the subthalamic nucleus (STN) varies from atlas to MRI, as well as between patients, the target marker has been placed in a position, which would be deemed as adequate lead placement. To the left: the location and size of the caudal zona incerta (cZi) on the level of Z = -3.5 mm. Based on the Atlas for Stereotaxy of the Human Brain by G.Schaltenbrand and W.Wahren, 1977 © Thieme. To the right: T2-weighted MRI at the level of the maximum diameter of RN.

The surgical technique has been described in detail previously^19,33^. The operations were frame-based stereotactic implantations of the DBS electrode 3387 or 3389 (Medtronic, Minneapolis, MN, USA) targeting the cZi. The target was identified on stereotactic transaxial T2-weighted MR images. The cZi was defined as laying slightly postero-medial to the visualized posterior tail of the subthalamic nucleus (pSTN) at the level of the maximal diameter of the red nucleus^54^, see Fig. 4 (also “Targeting the thalamic/subthalamic area for tremor” by P. Blomstedt at https://stereotactic.org/). The entry point was planned to be on top of a gyrus within 10 mm of the coronal suture. The trajectory was planned as steep as possible without penetrating the ventricles and ensuring electrode localization medial to the STN.

The awake procedures were performed with intraoperative macrostimulation, and the depth of the electrode was, when indicated, optimized in accordance with the stimulation response. Microelectrode recording was not performed. After confirmation of the physiological target, the electrode was positioned with at least one contact located deeper than the intraoperative target. This was done to minimise the consequences of possible deviation due to brain shift.

In asleep procedures, Propofol-, Remifentanil- and Phenylephrine-infusion were used for induction and maintenance of general anaesthesia. Bolus injections of Rocuronium bromide was used as a muscle relaxant. After induction, the patients head was shaved and a Leksell stereotactic G frame (Elekta Instruments AB, Stockholm, Sweden) was mounted on the patient’s head before a stereotactic MRI was performed. A video demonstration of the surgical technique is available online (https://www.youtube.com/watch?v=89GYugaCt_I, “Implanting a DBS-system” from https://stereotactic.org). The surgical dressing was applied in a manner to allow for visible access to the area behind the ear as well as above and below the clavicle. The patients stayed in the same prone position throughout surgery and during electrode implantation the deepest contact was simply placed at the anatomical target as indicated above. After electrode implantation, with the frame still on the patient, a small incision was created behind an ear. A few cm large incision was created inferior to the ipsilateral clavicle and a subcutaneous pocket was created. By bending a tunnelling tool to accommodate for the patient’s neck anatomy, we were able to tunnel the extension cables with the frame still attached to the patient. A post implantation CT was performed, and the images were fused with the preoperative MRI for identification of the electrode location. The extension cables and implantable pulse generators (Models: Kinetra/Soletra/Activa SC/PC, Medtronic, Minneapolis, MN, USA) was implanted in the same session in all patients except one patient in the awake group.

### Evaluation

Four weeks after surgery, a monopolar review was performed, and each individual electrode contact was evaluated concerning the location and effects of stimulation. The contacts displaying the best effect in the absence of side effects were chosen for chronic stimulation. The patients were evaluated according to the Essential Tremor Rating Scale (ETRS)^58^ before, and one year after surgery, on and off stimulation. In patients with bilateral electrodes, body sides were evaluated separately (with the other side off stimulation) and are here reported separately. The stimulation was turned off during the night prior to off-stimulation assessment and turned on for at least 60 minutes prior to on-stimulation assessment.

### Contact localization

The locations of the active cathodic contacts and most distal contacts were derived from fused MRI-CT scans in all patients. The most distal contact was used as a surrogate measure of electrode placement. The distance to the mid-commissural point (MCP) and posterior tail of the STN (pSTN) was calculated for each contact using Cartesian coordinates (X,Y,Z). In cases where two contacts were activated, the mean coordinates of the two contacts were used for that electrode. The mean contact locations in relation to the pSTN of the different groups (awake and asleep) were visualized on serial slides of Morel's atlas with the borders of the boxes representing the standard deviation.

### Statistics

Statistical analysis was performed using IBM SPSS Statistics for Windows, Version 28.0 (Armonk, NY: IBM Corp). Mann-Whitney U test was used to compare ordinal values (ETRS scores) between groups and Wilcoxon signed-rank test for within-group analyses of pre- and postoperative ETRS scores. Pearson’s Chi-square test was used for comparison of categorical values. Welch’s T-test was used for comparison of continuous variables (age, disease duration, stimulation parameters and coordinates) between groups accounting for possible unequal variances and unequal sample sizes. Differences in variance of electrode location, as measured by coordinates of the deepest contact, was assessed using Levene’s test as a standalone analysis. Two-sided tests were used. A *p* value < 0.05 was considered statistically significant.

## Data Availability

The data that support the findings of the current study is available from the corresponding author upon reasonable request. Sharing restrictions will be applied to sensitive data to preserve the personal integrity of the participants.

## Code availability

Codes are available upon reasonable request to the corresponding author.
